# Longitudinal Outcomes: Student Perceptions of a Third-Party-Resource-Based Pre-matriculation Module

**DOI:** 10.7759/cureus.71297

**Published:** 2024-10-12

**Authors:** Kim Chosie, Sahil Bharwani, Stacey Jackson, Robyn Schwartz, Dominic J Gigliotti, Bridget Budny

**Affiliations:** 1 Medical Education and Research, Alabama College of Osteopathic Medicine, Dothan, USA; 2 Internal Medicine, Oschner Medical Center, Jefferson, USA; 3 Pediatrics, Norwell Health, New York City, USA; 4 Urology, St. Joseph's Medical Center, Stockton, USA; 5 Medicine, Virginia Commonwealth University Health System, Richmond, USA; 6 Surgery, Naval Medical Center, Portsmouth, USA

**Keywords:** alabama college of osteopathic medicine, medical school, pre-matriculation, resources, student success

## Abstract

Medical school is grueling. Students are required to learn large amounts of subject matter in short periods of time, while also participating in extra-curricular activities to become well-prepared and future residents. While many third-party resources can aid students in navigating the curriculum, the process of assessing and selecting these tools can be time-consuming and frustrating. To address this challenge, a pre-matriculation module was developed to introduce students to various third-party resources, enabling them to identify their preferences and understand their learning styles before matriculation. Links to free trials were also provided. Additionally, a supplement to the module featured advice and strategies from top-quartile students (now Doctors of Osteopathic Medicine (D.O.s)) on how to succeed in the pre-clinical curriculum.

The module included voice-over slide presentations that highlighted third-party resources, tips for using these resources effectively, and insights from contributors on succeeding in the pre-clinical phase. To assess the perceived efficacy of the program, surveys were administered to incoming first-year osteopathic medical students (OMS-1) students at several time points: before beginning the first semester and pre-matriculation module, immediately after completing the module, and at the end of the first semester. Further surveys were conducted at the end of the second, third, and fourth semesters. Statistical analysis of the survey data was performed to evaluate students’ perceptions of the module’s value.

Results indicated that students perceived the pre-matriculation module as significantly contributing to their pre-clinical academic success. These findings suggest that early exposure to third-party learning resources and tailored guidance from successful peers may ease the transition into medical school and foster better academic outcomes. Future research should explore the long-term impact of such pre-matriculation interventions on clinical performance and overall medical education. Additionally, investigations into tailoring modules to different learning styles or specific specialties could further optimize the student experience and success in medical education.

## Introduction

Medical school is difficult primarily because of the amount of dense material students must learn and remember in a relatively short period of time while pursuing all of the extra-curricular activities necessary to be competitive in the residency match process. The American Medical Association [1] discusses the benefits of using third-party resources in an effort to help students master the volume of material they must learn and retain in a very short timeframe. They reference a "2022 Association of American Medical Colleges Year 2 Survey that reported approximately 70% of medical students used non-institution-created online videos and other online content daily or weekly for medical education information" [2,3]. A qualitative study of pre-clinical medical students' attitudes about third-party resources found that students praise them for their content, or high-yield information as well as their clarity and efficiency [3]. While the literature shows that third-party resources can help students succeed once they are established in the curriculum, having the time to assess and choose the ones that fit a student's needs can be frustrating and take valuable and limited student time [1,2]. For this reason, a pre-matriculation module was created to expose students to third-party resources so they could determine in advance which they prefer as well as understand their learning style. Links for free trials were included in the module. A supplement to the module included advice and suggestions from successful (top-quartile) students (now osteopathic physicians) on how to succeed in the pre-clinical curriculum with samples of their study processes.

## Materials and methods

The modules contained voiced-over PowerPoint Presentations (Microsoft Corporation, Redmond, USA) that shared third-party resources most commonly used at the Alabama College of Osteopathic Medicine (ACOM), how to use those resources effectively, and opinions of the contributors on how to succeed in the pre-clinical curriculum. The modules were created by then 2nd- and 3rd-year osteopathic medical students and the Director of Academic Support Services. Each module covered a specific third-party resource, Anki for example (Digital Dream Labs, Pittsburgh, USA), with sample questions and specific steps on how to use the resource. Links for free trials of each resource were offered when possible as well. The Director of Academic Support created a module on effective methods of studying in medical school as well as how students could identify their learning style and match it to the proper resource. Included in the module were institutional resources (library, lib-guides, videos, etc.) as well as mental and physical health resources.

Students were given access to the module prior to matriculation and it was added to their learning management system so they would have access to it for the entirety of their pre-clinical curriculum (four semesters). They were encouraged to view the module prior to matriculation, access free trials of the resources using the links provided and become familiar with them allowing students to choose which resources they thought would be helpful or that aligned with their learning style prior to the presentation of curriculum.

To assess students' perceived efficacy of the module, surveys were sent in each semester of the preclinical curriculum. One was sent prior to beginning the first semester and prior to beginning the module, the second survey was sent immediately after completing the module, and the third survey was sent at the end of the first semester. Surveys were also sent at the end of the 2nd, 3rd and 4th semesters. The data was then statistically analyzed to determine the students’ perceived value regarding the module. 

The study started with 186 participants in semester 1 and ended with 55 participants in semester 4. The study began on Jul 25, 2022, and ended on June 30, 2024, covering four pre-clinical semesters at ACOM (Table 1).

**Table 1:** Study demographics

Semester	Participants (Start)	Participants (End)	Dropout Rate (%)
Semester 1	186	186	0%
Semester 2	186	135	27.42%
Semester 3	135	94	30.37%
Semester 4	94	55	41.49%

Criteria required for inclusion in the study were that the participants had to be pre-clinical medical students enrolled at ACOM. Participants were given access to the pre-matriculation module prior to the start of their first semester and had to complete at least one survey during any of the four pre-clinical semesters. Participants had to have access to the third-party resources included in the module, as well as the provided free trials. Only students who consented to participate in the study were included in the final analysis.

Criteria for exclusion from the study were non-ACOM students and students who did not complete any of the surveys were excluded from the final analysis. Students who left ACOM during the pre-clinical years for academic or personal reasons were excluded from the final analysis as were students who could not access the free trials or were unable to engage with the pre-matriculation module were excluded from the study.

Statistical tools used for data analysis were the Mann-Whitney U Test which is a non-parametric test, used to compare differences between two independent groups, specifically between pre-module and post-module survey responses regarding students’ perceived preparedness for medical school. The Kruskal-Wallis One-Way ANOVA non-parametric test was used to compare perceived resource effectiveness across more than two groups (e.g., different semesters), followed by Dunn’s correction to adjust for multiple comparisons. Finally, a Two-Way ANOVA test was used to assess changes in resource utilization and student feedback over time, specifically to examine trends in resource use (such as Anki or UWorld (UWorld, LLC, Dallas, USA)) over the four semesters. Tukey's multiple comparisons test was used post hoc to determine significant differences between semesters.

## Results

The results of this study show that the pre-matriculation module, aimed at familiarizing incoming medical students with third-party resources, was highly valued by students and perceived to significantly contribute to their academic success throughout the pre-clinical curriculum. Key findings include:

Students reported feeling more confident and better prepared for medical school after completing the module. Survey results indicated a significant shift in students' perceived readiness between the pre-module and post-module periods. This preparedness extended beyond knowing which resources to use and included a better understanding of how to match resources to their learning styles (Table 2).

**Table 2:** Student responses pre/immediate post-survey regarding their preparedness for medical school See Appendices for the Pre-Survey document.

	Mean	3.02
Pre-Survey	Std. Error of Mean	0.077
	N	162
	Mean	3.37
Immediate Post-Survey	Std. Error of Mean	0.102
	N	73
	Mean	3.12
Post-Survey Responses	Std. Error of Mean	0.063
	N	237

Throughout the pre-clinical years (semesters 2-4), students consistently used third-party resources. Popular resources like Anki, Sketchy (Sketchy Medical, Irvine, USA), and question banks (e.g., UWorld) saw increased utilization over time. Anki, known for its focus on active recall and spaced repetition, became one of the most frequently used tools, with its use rising each semester. Other resources such as Pathoma (Pathoma, Lincolnwood, USA) and Sketchy were also widely adopted due to their high-yield content and board relevance. Interestingly, while YouTube videos initially saw significant use, this trend decreased slightly from the third to the fourth semester, suggesting a shift toward more structured, paid resources as students advanced in their studies (Table 3).

**Table 3:** Resource utilization

	Pre-Survey (N=186)		Immediate Post Survey (N=186)		Semester 2 (N=135)		Semester 3 (N=94)		Semester 4 (N=55)	
Resources	Frequency (N)	Percent %	Frequency (N)	Percent %	Frequency (N)	Percent %	Frequency (N)	Percent %	Frequency (N)	Percent %
Anki	162	87.1	69	37.1	135	100	64	68	44	80
Sketchy	96	51.6	59	31.7	98	72.5	50	53.1	40	72.7
YouTube Videos	155	83.3	57	30.6	12	8.8	72	76.5	43	78.1
Question Banks	154	82.8	64	34.4	99	73.3	75	79.7	55	100
Pathoma	69	37.1	42	22.5	96	71.1	56	59.5	45	81.8
Other	23	12.4	4	2.1	48	35.5	37	39.3	32	58.1

Feedback from the surveys conducted at various points throughout the students' pre-clinical years consistently showed high ratings for the perceived usefulness of the pre-matriculation module. Students expressed that the module provided them with a framework for approaching medical school, reducing anxiety and saving time that would otherwise be spent searching for effective study resources during an already stressful academic period. By the end of the first semester, the majority of students reported that they had already identified which resources worked best for them, attributing this advantage to the pre-matriculation module

The module's inclusion of advice and strategies from top-quartile students (now osteopathic physicians) resonated strongly with participants. Students appreciated the practical tips on how to navigate the pre-clinical curriculum and found the recommendations for high-yield resources particularly helpful. The success strategies offered by more advanced students reinforced the use of heuristics-mental shortcuts like "imitating the successful" or choosing familiar tools, which were instrumental in helping students make quick, effective decisions about resource selection (Table 4).

**Table 4:** Supplemental resource utilization

	Semester 2 N=208		Semester 3 N=94		Semester 4 N=60	
	Frequency (N)	Percent(%)	Frequency (N)	Percent(%)	Frequency (N)	Percent(%)
Yes	154	74	69	73.4	54	90
System	54	26	25	26.6	6	10
Total	208	100	94	100	60	100

One of the primary benefits identified in the results was the significant reduction in time spent by students evaluating and selecting third-party resources during their medical education. Students reported that having access to free trials and guidance on how to use the resources before starting medical school alleviated much of the pressure associated with deciding which tools to invest in. By entering their pre-clinical years with a solid understanding of available resources and their personal learning preferences, students were able to focus more on content mastery rather than on navigating resource options. The positive perceptions of the module's impact on academic success were sustained throughout the entire pre-clinical curriculum (semesters 2-4). As the curriculum became more challenging, students continued to rely on the guidance and resources provided in the module, reaffirming its long-term value.

Despite the overwhelming positive feedback, some students raised concerns about the cost of third-party resources, particularly given the already high tuition fees associated with medical school. The module's provision of free trial links was appreciated, but students noted the financial burden that comes with continuing to use some of these resources beyond the trial period. Another challenge highlighted by the study was the sheer number of available resources, which, despite the module's guidance, could still feel overwhelming to some students.

The study concludes that the pre-matriculation module had a strong positive impact on students' academic performance and resource management throughout the pre-clinical curriculum. By providing early exposure to third-party resources and allowing students to experiment with these tools in a low-pressure environment, the module helped students develop personalized study strategies that aligned with their individual learning styles.

Future research will focus on correlating the use of these third-party resources with objective academic outcomes, such as COMLEX Level I and Level II first-time pass rates. Tracking student performance across these critical exams will help determine if early exposure to and effective use of these resources leads to measurable improvements in exam outcomes. Additionally, further surveys will be conducted to assess whether the benefits observed in the pre-clinical years translate to success in clinical rotations and licensing exams.

## Discussion

Medical students routinely use third-party resources because of the perceived benefits of using them versus using resources provided by their medical school [4]. Their desire to find a way to be successful given the burden of an overwhelming amount of material to be mastered created an often frustrating decision-making process when it came to selecting which resources to use. They often chose resources dependent on others' advice and in situations that helped them save time, and while navigating time and financial constraints. The anxiety to perform well in medical school to be competitive for residency led participants to focus on materials that they perceived helped them achieve this goal. These patterns align with the concept of bounded rationality and fast-and-frugal heuristics [5]. "Fast-and-frugal heuristics are part of one’s 'adaptive toolbox' and employ a minimum of time, knowledge, and computation to make adaptive choices in real environments" [6]. Students at the Alabama College of Osteopathic Medicine (ACOM) chose their resources based on the recommendations of more advanced, successful students, and those often included. The resources that emphasized active recall (Anki, Sketchy, Quizlet (Quizlet, San Francisco, USA), etc.) were the most recommended by the student authors due to their high-yield concepts and time-effective presentation. "Students used multiple heuristics to make choices, including the recognition heuristic (preference for the familiar), imitate-the-majority, and imitate-the-successful" [7]. "While heuristics as mental shortcuts are subject to biases, they ease decisions when time is limited and information incomplete" [5]. The ability to make fast decisions about what might be effective appears to make more sense than trying to learn every detail about every available resource. Many students described that rather than trying to discern the best resource to use for a topic, they would choose a resource that met their needs [5]. Even from this perspective, choosing proper third-party resources was difficult and time-consuming given the number of resources available and the weight of the outcome of using each resource.

The module was perceived by students at the Alabama College of Osteopathic Medicine (ACOM) as a valuable tool that improved their confidence and preparedness for medical school. It allowed students to navigate the often overwhelming landscape of study resources more effectively by helping them identify and choose tools suited to their individual learning styles. The consistent feedback from students across the four semesters demonstrates that early access to structured guidance and third-party resources plays a critical role in reducing the time and stress associated with resource selection. This proactive approach also helped students focus more on mastering content than on searching for tools, contributing to their overall academic success.

One of the key strengths of the module was its inclusion of advice from top-quartile students, which resonated strongly with participants. By offering insights from students who had successfully navigated the pre-clinical curriculum, the module not only provided concrete recommendations but also empowered students to use heuristics such as “imitate-the-successful” in their decision-making process. These mental shortcuts helped students make quick, effective choices about which resources to invest in, a particularly valuable strategy given the time constraints and academic pressures of medical school. Popular resources like Anki, Sketchy, and question banks (e.g., UWorld) were praised for their efficiency and relevance to board exams, reinforcing their status as essential tools for medical students.

While the module was perceived as highly effective, this study has several limitations that should be acknowledged. First, the reliance on self-reported data introduces the potential for bias, as students’ perceptions of their preparedness and resource utility may not accurately reflect their actual academic performance [8,9]. Additionally, the study did not measure objective academic outcomes, such as exam scores, limiting the ability to directly correlate the module’s perceived benefits with tangible improvements in academic success [10]. The high attrition rate by the fourth semester also raises concerns about the generalizability of the results, as the smaller sample size may not fully represent the broader student body’s experience. Furthermore, the module was designed specifically for students at ACOM, and its applicability to other institutions with different curricula, student demographics, or institutional cultures remains unclear. Finally, the cost of third-party resources, despite the inclusion of free trials, was noted by some students as a burden, which could impact the long-term sustainability of resource usage. Future research should address these limitations by incorporating objective performance metrics, controlling for external variables such as prior academic background, and expanding the study to include students from other medical schools.

## Conclusions

This study demonstrated that a pre-matriculation module aimed at exposing medical students to third-party resources significantly contributed to their perceived academic success during the pre-clinical curriculum. By offering early access to high-yield resources and practical advice from top-performing students, the module helped alleviate the anxiety and time burden associated with choosing effective study tools. Students reported increased confidence, better preparedness, and efficient resource selection, all of which supported their academic journey in medical school. These findings highlight the potential of structured pre-matriculation interventions to enhance medical education outcomes, particularly in environments where time management and resource selection are critical.

However, the study's reliance on self-reported data and lack of objective performance metrics limits the ability to directly link the module to improved academic outcomes. Future research should focus on correlating the module’s usage with measurable academic achievements, such as COMLEX Level I and II first-time pass rates. Additionally, expanding this research to other medical institutions can determine the generalizability of the module’s effectiveness. Investigating the long-term impact of pre-matriculation guidance on clinical success and board exam performance will also provide valuable insights for improving medical education practices.

## Figures and Tables

**Figure 1:**
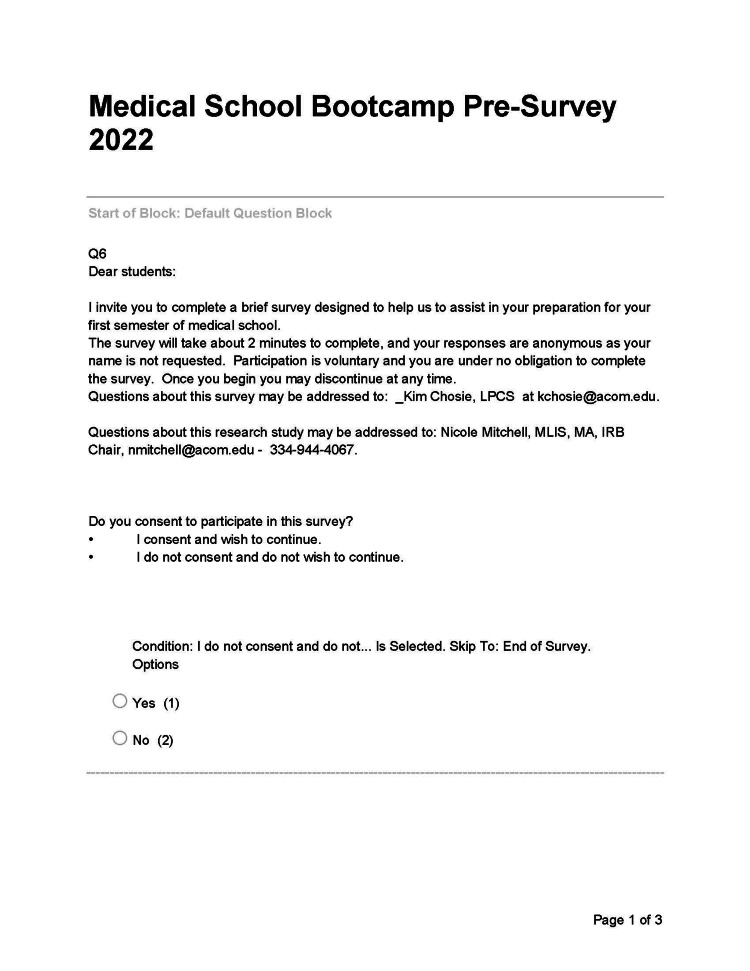
Medical School Bootcamp Pre-Survey Page 1

**Figure 2:**
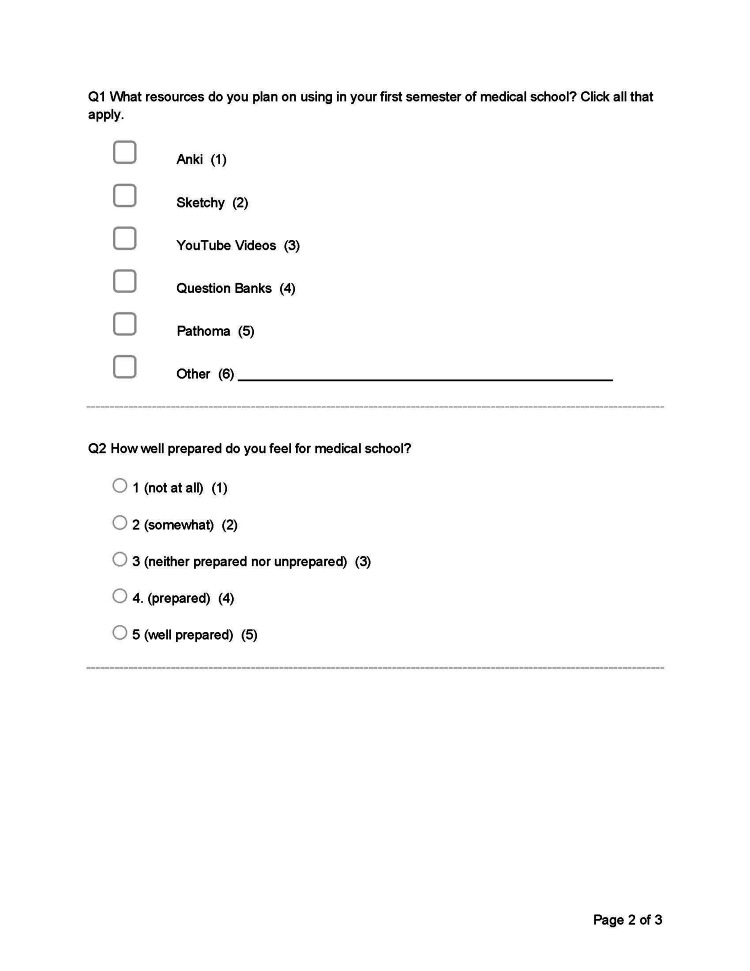
Medical School Bootcamp Pre-Survey Page 2

**Figure 3:**
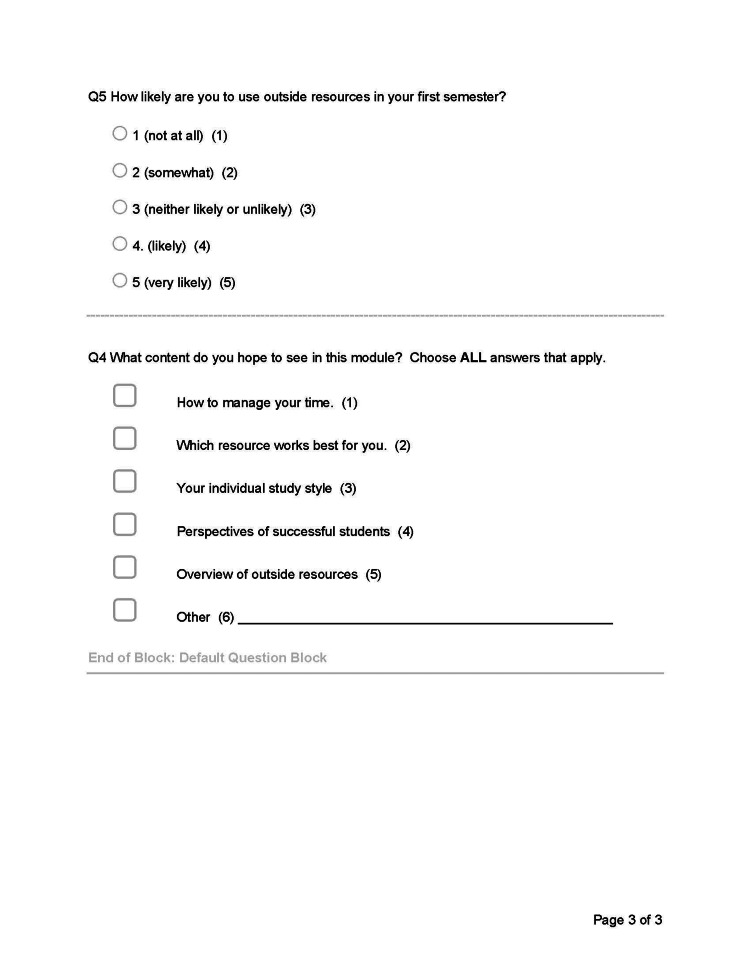
Medical School Bootcamp Pre-Survey Page 3
